# Language and/or memory: How to slice the domain-cake?

**DOI:** 10.1080/02643294.2025.2535037

**Published:** 2025-07-20

**Authors:** Vitoria Piai, Christopher R. Madan, Jolien C. Francken

**Affiliations:** a Donders Institute for Brain, Cognition and Behaviour, Centre for Cognition, Radboud University, Nijmegen, Netherlands; b School of Psychology, University of Nottingham, Nottingham, UK; c Swammerdam Institute for Life Sciences, University of Amsterdam, Amsterdam, Netherlands

**Keywords:** cognitive ontology, episodic memory, hippocampus, word retrieval, recall, retrieval

## Abstract

Historically, memory and language have been seen as separate cognitive functions and studied in isolation. To date, it remains an open question to what extent these cognitive domains are related. Here, we present the interdisciplinary discussions from the 42nd European Workshop on Cognitive Neuropsychology around the central question of how we should see the relationship between the domains of language and memory. We discuss relevant empirical evidence from the fields of cognitive psychology, cognitive neuropsychology, and cognitive neuroscience and take a philosophical perspective on this central question, considering issues such as how to weigh different types of evidence and how to conceptualize the relationship between language and memory. We conclude that elucidating questions about the nature of the relationship between language and memory requires not only more empirical data, but also parallel conceptual development.

## 1. Introduction

In its 125th anniversary issue, the journal *Science* asked 125 fundamental scientific questions, among which “How are memories stored and retrieved?” (Kennedy & Norman, 2005). Broadly defined, memory is “a compendium of useful and trivial facts about the world, the history of our lives, plus every skill we’ve ever learned, from riding a bike to persuading a loved one to take out the trash” (Miller, 2005, p. 92). The retrieval of words from memory is fundamental to humans’ unique ability to use language and may be argued to depend on evolutionarily older mechanisms, like those utilized by (“nonlinguistic”) memory. However, historically, memory and language have been seen as separate cognitive functions and studied in isolation.^1^ This divide perhaps has some roots in striking neuropsychological cases, starting as early as the 1860s with descriptions by Gustave Dax and Paul Broca of the loss of articulated language (Levelt, 2013), and in the 1950s by Scoville and Milner on the loss of recent memory (Scoville & Milner, 1957).

Still today, many (formal and informal) references to the domain of memory tend to exclude the domain of language and vice-versa, or at least do not take an explicit stance on the relation between these two cognitive domains. Yet neglecting the relationship between these two domains hinders cross-domain knowledge transfer, which is important for achieving a unified theory of brain and cognition. Moreover, integration across domains has the potential to increase impact on clinical and applied settings (e.g., Eikelboom et al., 2018; Middleton et al., 2020; Morrow & Duff, 2023; Ullman & Lovelett, 2018).

Despite these reasons for studying the relationship between the domains of memory and language, it is still an open question to what extent these domains are related. The central question in this paper is how we should slice the domain-cake, i.e., how to see the relationship between the domains of language and memory.

In the literature, different methodological approaches are used in order to answer this central question. For example, single case studies have been powerful for demonstrating that the ability to retrieve memory episodes is independent of the ability to retrieve words (e.g., Kensinger et al., 2001), and the other way around (e.g., Basso et al., 1988). A different methodological approach consists of comparing brain regions that are activated during both retrieval of episodic and linguistic information. For example, if the active areas overlap, one tends to conclude that the two types of retrieval share neural resources, and hence language and memory should be regarded as interrelated domains. Conversely, one tends to conclude that they are distinct domains if the corresponding configuration of neural activation does not show spatial overlap (e.g., reviewed for episodic versus semantic memory more broadly defined in Renoult et al., 2019).

### 1.1. European Workshop on Cognitive Neuropsychology Symposium

The work presented here was discussed during the 42nd European Workshop on Cognitive Neuropsychology (EWCN), held in Bressanone, Italy, 21–26 January 2024. Our interdisciplinary symposium included talks by three speakers (the authors) coming from different (sub-)fields, namely cognitive neuroscience of episodic memory, cognitive neuroscience and neuropsychology of language, and philosophy of neuroscience. The first two speakers (VP, CRM) were asked to present a concise overview of particular aspects of the empirical literature on cognitive psychology, cognitive neuropsychology, and cognitive neuroscience that they found sketched a picture of how researchers aim to tackle this central question. The third speaker (JCF) was asked to present a perspective from (neuro)philosophy on cognitive ontology. Finally, the three speakers discussed, together with the audience, the issues that arise once one tries to answer the question of how to see the relationship between the domains of language and memory. Here, we present a synthesis and elaboration of the short symposium presentations and subsequent discussions that took place during the meeting. Given that this paper faithfully reflects the symposium presentations, we will not attempt to extend the empirical literature beyond what the speakers presented during the symposium (Sections 2–5). We do, however, go beyond the discussions during the symposium in that we critically analyse key issues related to the central question on the relationship between the domains of language and memory (Section 6).

**Figure 1.**
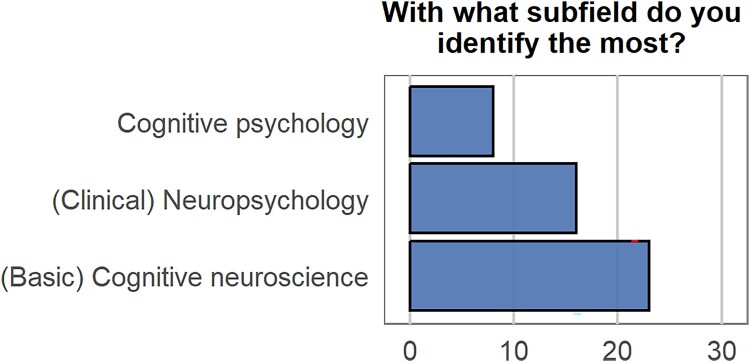
Poll response counts for each option for the prompt: “With what subfield do you identify the most?”. [To view this figure in colour, please see the online version of this journal.]

During the symposium, we conducted an informal survey^2^ with various poll questions related to the topics discussed in this paper and we will refer to the results of this survey in different sections. We emphasize that this survey was informal and these data should not be interpreted too strictly, but rather serve to give us a feeling about what attendees intuitively think. In total, 89 participants responded (but not all to all questions). We first inquired with what subfield the participants in the audience identified the most. Figure 1 depicts the distribution of respondents across the different fields we included as answer options (forced choice). As is evident from the figure, the symposium was also interdisciplinary in its audience.

### 1.2. Present article

For the sake of fruitfulness, the symposium focused on specific cognitive (sub)functions within each domain, namely those of (episodic-)memory retrieval and word retrieval. Here, we define episodic-memory retrieval as being able to produce (i.e., recall) information from memory (e.g., a word, an image, etc.) as having been presented within a recent episodic context (e.g., such as in tasks like free recall and cued recall, defined further in Section 2). Here, we define word retrieval as to include the retrieval of conceptual (meaning), lexical (abstract labels), and phonological (sound) information (together termed “linguistic information” henceforth), and the mapping between these aspects, for both word comprehension and word production. In this paper, it will be argued that although these initial definitions seem to be strict, they do not suffice, because first, ultimately it depends on how these cognitive functions are operationalized (see Section 5.3), and second, researchers do sometimes not adhere to their initial definitions.

In this paper, we will discuss the relevant empirical evidence presented during the symposium by each speaker. However, in order to answer the “domain-cake question” about the relationship between language and memory, we argue that bringing empirical evidence to the table is not sufficient. Therefore, we will also reflect on theoretical or philosophical questions such as how to weigh different types of evidence, and how to conceptualize the relationship between language and memory.

This paper is structured as follows. In Sections 2, 3, and 4, we review several empirical studies presented during the symposium. In Section 5, we present a philosophical viewpoint on the central question of how to slice the domain-cake or how to see the relationship between the domains of language and memory. In Section 6, we analyse and discuss the main issues that arise when attempting to answer this question, and propose directions for how to make progress. Here we also critically discuss the empirical evidence presented in Sections 2–4 in light of these recommendations. We highlight the strength of an interdisciplinary perspective in critically evaluating the questions themselves. We conclude that, besides making implicit assumptions explicit, we also need to define a-priori and explicitly the kind of relationship that we seek to understand.

## 2. Cognitive psychology

Within the memory literature there is a tradition of using verbal material. Extending from this, some word properties—such as word frequency and imageability—have long been studied for their effects on memory. These studies provide one perspective for studying interactions between language and memory, where the memoranda systematically vary in specified lexical properties and we examine the resulting memory performance and make inferences about the correspondence to memory-related cognitive processes.

**Figure 2.**
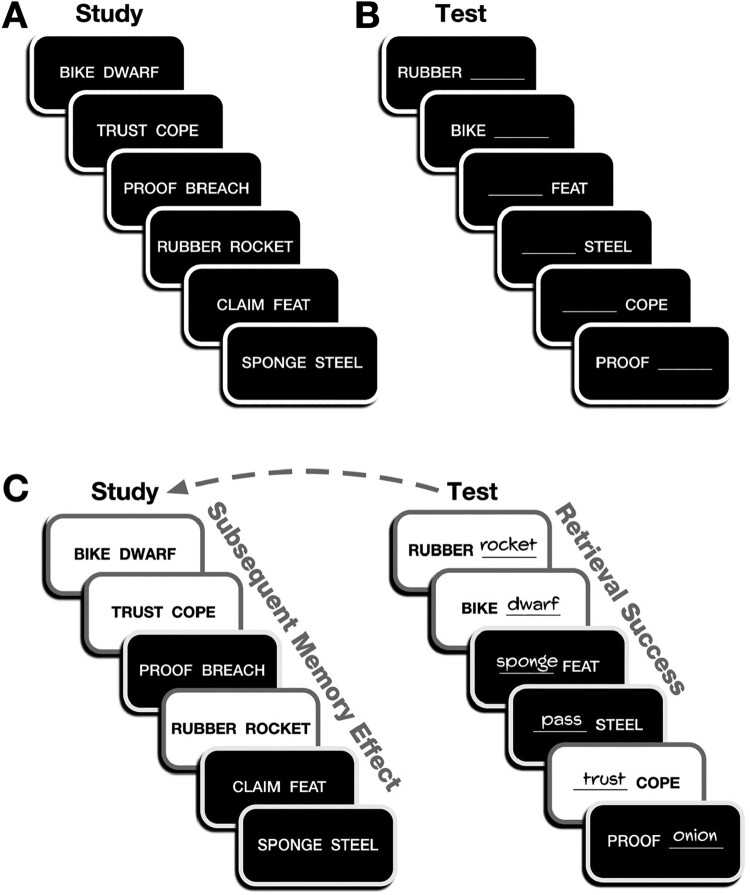
Illustration of task procedure. (A) Verbal paired-associates learning, (B) cued-recall test, (C) classification of trials for retrieval success and subsequent memory effects. In panel C, white boxes highlight correct trials and black boxes denote incorrect responses. Figure reprinted from Madan (2024) with permission.

A set of cued-recall studies can provide an example of this in action. In verbal paired-associate learning, participants typically intentionally learn novel (i.e., episodic) associations between pairs of words. When tested using cued recall, one word is provided as the cue—with the participant tasked with recalling the associated word, the target. When both words are high frequency (i.e., common words), accuracy is higher than when both words are low frequency (Clark, 1992; Clark & Burchett, 1994). As examples, high-frequency words include AREA and QUIET; low-frequency words include ANNEX and HOOT. Similarly, accuracy is better for high-imageability word pairs (i.e., words for referrents that are easily imagined; e.g., BIKE and ROCKET) than for low-imageability word pairs (e.g., COPE, TRUST) (Lockhart, 1969; Paivio, 1965, 1968; Wood, 1967). From this one might conclude that both properties similarly influence memory. However, we could include associations that are a mix of high-frequency and low-frequency words, e.g., AREA-HOOT. Moreover, cued recall can be tested in two testing directions, depending on which item is provided as the cue (Kahana, 2000; Mason et al., 2024). An illustration of this procedure is shown in Figure 2A,B.

**Figure 3.**
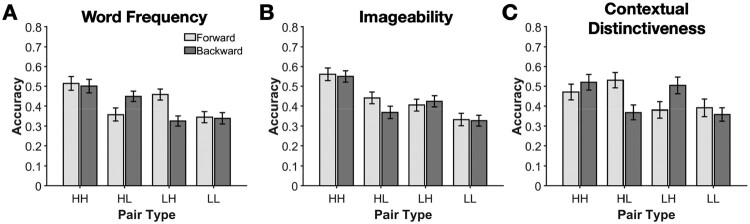
Cued-recall accuracy for each manipulated word property. (A) Word frequency, (B) imageability, and (C) contextual distinctiveness. Pairs of words were either both high for the word property (HH), a mixed high-low pair (HL), low-high, (LH), or both low (LL). Word pairs were tested in both the forward and backward test directions. Data for panels A and B are from Madan et al. (2010, Exp. 1); panel C from Criss et al. (2011, Exp. 4).

Cued-recall success can be thought of as a series of memory processes that all need to occur successfully to result in the correct target recall: cue recognition, the episodic association between the “fuzzy” representation of the two items, and target retrieval (Adams & Bray, 1970; Madan et al., 2010, 2012, 2020; McGovern, 1964). For instance, failure at this last stage could be considered as having the word on the 'tip of the tongue'. Madan et al. (2010) examined how word frequency and imageability influenced cued-recall performance and included mixed pairs, test direction, and considered the involvement of these three processes. Recall for pure pairs varying in word-frequency and imageability replicated prior work. In word frequency, recall for mixed pairs varied in relation to the target word: Recall was better when the target word was high frequency, as shown in Figure 3A. In contrast, for imageability, recall for mixed pairs was at an intermediate level, relative to the pure high and low pairs, and did not depend on which word was the cue or target (Figure 3B). Another study had similar goals and examined word frequency and contextual distinctiveness (Criss et al., 2011). Despite procedural differences, cued-recall performance for word frequency was comparable. Context distinctiveness is the uniqueness of a word to a specific context; as examples, COLT and RETINA appear in few contexts, whereas HOBBY and TALENT occur in more varied contexts. In mixed pairs, the cue primarily dictated success; recall was better when the cue was high in context distinctiveness, comparable to the pure high pairs (Figure 3C). Taken together, these findings suggest that word properties can influence how episodic associations are retrieved.

Providing further evidence of the role of language in memory, Madan (2021) examined the role of 20 properties in a large-scale free-recall study involving over 1500 words. Rather than manipulating word properties to be high or low for a specific property, a diverse selection of words was used that varied across many properties. Referent object size, animacy, and usefulness were the most related to free-recall probability. These attributes could be considered semantic knowledge—the interface of language and memory.

From this evidence, cognitive processes like word comprehension and word retrieval can be viewed as being heavily reliant on memory. There has been a tradition of using words as memoranda because they are useful *units* and their different properties influence how they are stored and accessed in memory. This perspective becomes even more apparent when considering the foreign language learning and alternate character scripts that can be used to communicate the same ideas (Madan, 2024, pp. 379–380; Pimsleur, 1967). Language provides labels for making sense of the world and remembering past experiences; varying word properties can be a useful tool for studying memory processes. However, language can be argued to be a domain of knowledge, just as we have specialized cognitive processes for faces and objects. As such, it could be argued that language is based in memory.

## 3. Cognitive neuropsychology

In populations with brain damage, the retrieval of linguistic information can be studied with a picture-naming task,^3^ whereby word retrieval performance is tapped into by the number of correctly named objects as well as the number of specific types of errors made during naming. Low accuracy in picture naming and, more specifically, incorrect responses, failures to name (e.g., “I don’t know” responses), and semantic errors are all indicative of disruptions during word retrieval.

In a study by Hilverman and Duff (2021), naming performance of three participants with bilateral hippocampal damage (two due to anoxia and one due to herpes simplex encephalitis) was compared to performance of ten neurologically healthy control participants (HC), matched on a number of relevant demographic variables. The three individuals had had profound anterograde amnesia for longer than 17 years, but no typical language, naming, or conceptual deficits as assessed through interviews with a speech-language pathologist and standardized neuropsychological batteries. Relative to the HC group, individuals with amnesia produced fewer correct responses, had more instances of “I don’t know” responses, and were less accurate when naming less familiar words.^4^

Whereas Hilverman and Duff (2021) examined word retrieval in a population with well-characterized episodic memory impairment (and found that word retrieval too is affected), the counterpart of this would be to examine episodic memory in a population with well-characterized language impairment. Eikelboom et al. (2018) performed a meta-analysis of verbal and nonverbal episodic memory tasks in individuals with primary progressive aphasia (PPA), a progressive language disorder. According to diagnostic criteria, language impairment is the most prominent symptom from the onset of symptoms, while prominent episodic and nonverbal memory impairment in the initial phase is an exclusion criterion (Gorno-Tempini et al., 2011). For the meta-analysis, episodic memory performance was operationalized as the score obtained from a neuropsychological test, according to clinical assessment standards, validated to measure episodic memory (e.g., California Verbal Learning Test, Philadelphia Verbal Learning Test, Rey Auditory Verbal Learning Tests, Rey’s Complex Figure Test, etc.). Performance was compared between PPA groups and groups of matched HC, both for verbal materials (i.e., digits, words, sentences, stories) and non-verbal materials, such as images of (abstract) objects and scenes. The authors found that performance in episodic memory tasks was significantly worse in the PPA groups compared to matched HC for both verbal and non-verbal materials.

Together, these neuropsychological results indicate an association between deficits in word retrieval and in episodic memory (albeit more pronounced in one domain than in the other within each population). If one can demonstrate that these associations are not driven by confounds or alternative explanations (e.g., Davies, 2010), these results would indicate that episodic retrieval and word retrieval have some kind of relationship, for example, that they depend on each other or, rather, that they both share an underlying cognitive mechanism or neural basis. Notably, it does not seem justified to draw stronger conclusions on the basis of the presented evidence, an issue that we will discuss in more detail in Section 6.

## 4. Cognitive neuroscience

**Figure 4.**
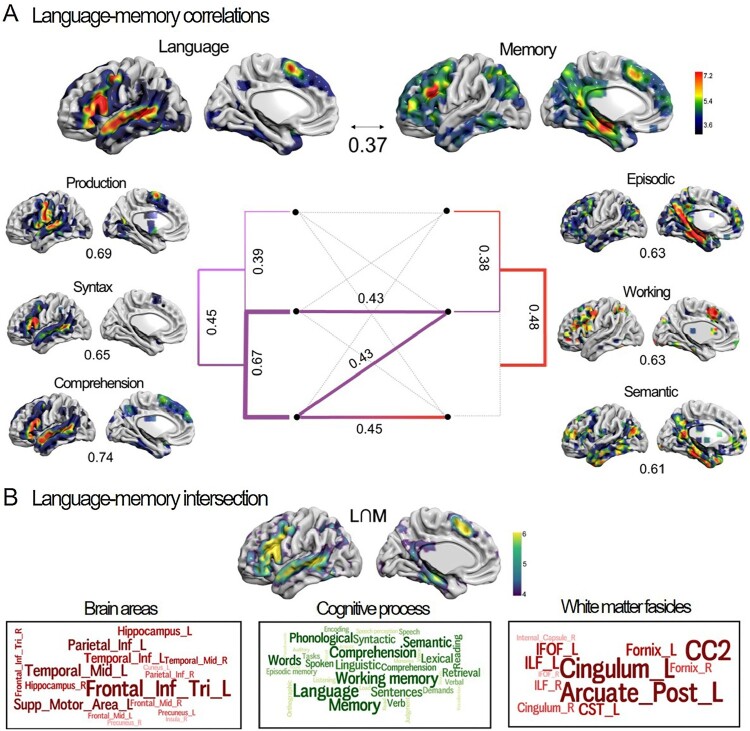
Brain regions involved in language and memory function. (A) Results from automated meta-analysis of fMRI studies (via NeuroSynth), showing meta-analytic brain activity functional maps for language, memory, and representative subfunctions of each. Correlations between maps are shown between the overall maps (*r* = 0.37), as well as for subfunctions in the tanglegram. (B) Brain regions, cognitive processes, and white matter fascicles associated with the language-memory overlap. Figure reprinted from Roger et al. (2022, Figure 3) with permission. [To view this figure in colour, please see the online version of this journal.]

More broadly, many brain regions can be labelled as being involved in both language and memory function. Using an automated meta-analysis of fMRI studies (via NeuroSynth), a recent review by Roger et al. (2022) examined the spatial overlap between language and memory processes (see Figure 4). The meta-analytic brain activity functional maps for language were examined, as well as three subfunctions: verbal production, syntax, verbal comprehension. For memory, the meta-analytic brain activity functional map for declarative memory was examined as well as for the subfunctions of episodic memory, verbal working memory, and semantic memory. Language and memory functional maps had a correlation of 0.37, with significant correlations between the subfunctions shown in Figure 4A. From this analysis approach, Roger et al. (2022) found that, consistent with prior literature, working-memory processes were at the intersection of language-memory function. To aid in interpreting the spatial overlap, Figure 4B shows the overlap between the language and memory functional maps, as well as word clouds representing the brain regions, cognitive terms, and white-matter fascicles that comprise the overlap.

The approaches discussed in Section 2 can also lead to cognitive neuroscience research questions and further the debate on language-memory interactions. A subsequent study asked how the hippocampus and other brain regions preferentially support the learning of imageable pairs (Caplan & Madan, 2016). Given the behavioural finding of enhanced episodic memory for novel associations between pairs of imageable as compared to abstract words, it is unclear how different brain regions support this outcome. For instance, do language regions, outside the medial temporal lobe, support episodic association learning through an automatic (interactive) imagery process? In a fMRI study, participants learned pairs of high- and low-imageability words and were tested using cued recall. Brain activity during study was examined for differences, related to both imageability and subsequent memory (see Figure 2C). Brain activity results suggested that the improved recall performance was not due to additional support or processing by regions outside the hippocampus, rather, imageability increased hippocampal engagement during study. This study evaluated a specific case of the role of language in enhancing memory and concluded that the effect was due to memory mechanisms, not language.

Whereas a common approach in the literature is to look for (lack of) overlap in activated brain regions between two tasks based on indirect brain measures such as those derived from fMRI, as exemplified above, it has also been disputed whether spatial overlap in itself is sufficient to determine that two domains should be regarded as one domain (e.g., Anderson, 2015; Piai & Zheng, 2019). More specifically, it can be argued that beyond their metabolic demands, neurophysiological responses produced by these areas, as measured through electrophysiology, should also overlap or show similarity. This question can be investigated through the use of the brain’s electrophysiology, which provides a direct measure of net neuronal activity.

**Figure 5.**
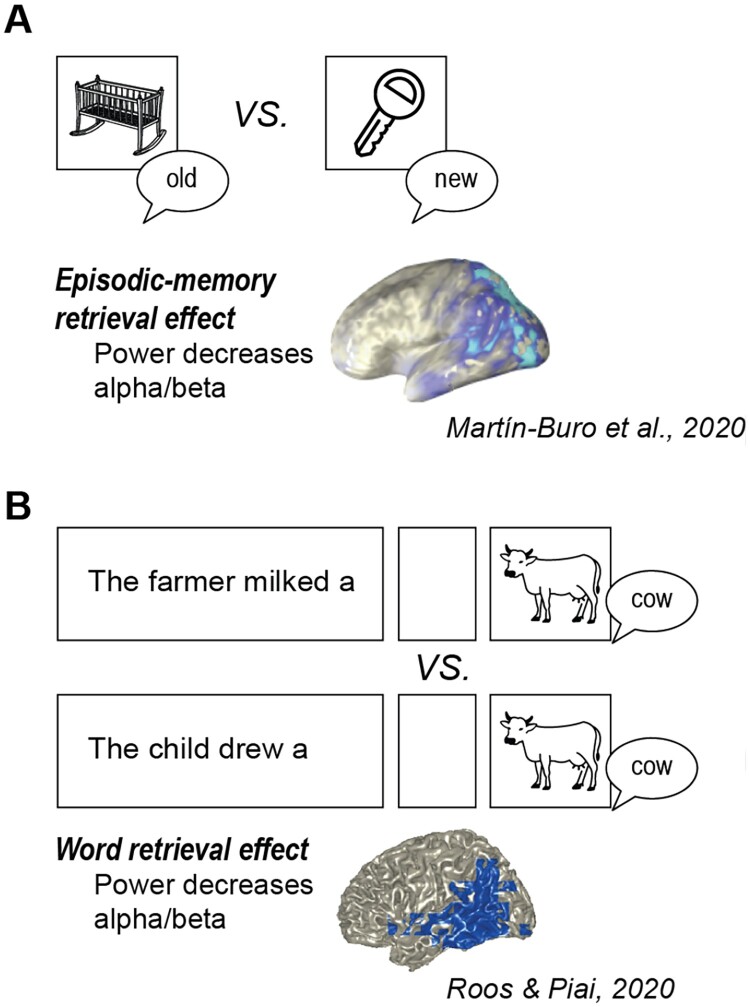
(A) Illustration of the task procedure for the episodic memory retrieval effect, with an old/new recognition task and source localization of the associated neurophysiological responses as power decreases in alpha/beta. Source map reprinted with permission from Martín-Buro et al. (2020, upper left portion of Figure 3). (B) Illustration of the task procedure for the word retrieval effect, with a sentence-primed picture naming task and source localization of the associated neurophysiological responses as power decreases in alpha/beta. Source map based on Roos and Piai (2020). [To view this figure in colour, please see the online version of this journal.]

Previous studies investigating retrieval (and encoding) in the episodic memory domain have made use, for example, of an old/new recognition test (see Section 2). The comparison between correctly identified old versus new items serves as a measure of episodic memory retrieval. Its associated neurophysiological responses are found predominantly as increases in power in medial temporal lobe structures between roughly 2–8 Hz (termed “theta”, see Jacobs, 2014), as well as decreases in power between roughly 8–25 Hz (termed “alpha" and "beta”), originating from lateral temporal and prefrontal structures (reviewed in Hanslmayr et al., 2016; Nyhus & Curran, 2010), with an example shown in Figure 5A.

The neurophysiological responses for word retrieval have been studied through sentence-primed picture naming. In the case of picture naming primed by a lead-in sentence, the amount of semantic information in the sentence can be manipulated in order to trigger the retrieval of linguistic information already prior to the picture (e.g., “The farmer milked a”, picture to be named: COW) versus not (e.g., “The child drew a”, picture to be named: COW). During the interval after sentence offset but before picture presentation, retrieval of linguistic information will take place in the primed condition but not in the unprimed condition, yielding a “word retrieval” effect (Figure 5B). Neurophysiological responses associated with word retrieval in this experimental context have also been found as decreases in power between roughly 8–25 Hz, originating from lateral temporal and prefrontal structures (Piai et al., 2015; Roos & Piai, 2020), with an example shown in Figure 5B, as well as power increases between 2 and 8 Hz from medial temporal lobe structures (Piai et al., 2016; Pu et al., 2020).

Ongoing studies (by Piai and collaborators) have been designed to directly test the similarity in neurophysiological responses between word retrieval and episodic memory retrieval. In those studies, we combined the word retrieval effect with the episodic old/new recognition effect (based on Perrone-Bertolotti et al., 2015). Participants performed the word retrieval task explained above (and illustrated in Figure 5B). After a short break, they were asked to do a recognition task on written words. The words could be “old words”, that is, words for the concepts they had previously named as pictures, and “new words” matched on relevant features to the “old words” (see also Section 2 above). In this way, the neurophysiological responses for word retrieval and episodic memory retrieval can be compared directly within participants (based on the same linguistic information).

Based on the results of the neurophysiological responses, we can claim that neuronal operations for retrieving linguistic information and those for retrieving information about episodes, as indexed by neurophysiological effects such as oscillatory power, are similar. Therefore, put strongly, the neuronal infrastructure people utilize for retrieving linguistic and episodic information is the same.

## 5. Philosophy of neuroscience

### 5.1 Cognitive ontology and the domains of language and memory

From a philosophy perspective, the question how language and memory relate bears on a broader question about our so-called “cognitive ontology”. The general aim of cognitive neuroscience is to map cognitive capacities to neural mechanisms, and to discover how parts and processes of the brain can explain parts and processes of the mind. However, to achieve that aim, we need to know *what* exactly are these cognitive capacities that we want to explain. Explicitly or implicitly, we use taxonomies of cognitive capacities, or cognitive ontologies (Anderson, 2015; Janssen et al., 2017; Klein, 2012; Poldrack, 2010; Poldrack et al., 2011; Price & Friston, 2005). For instance, in cognitive (neuro)science we assume that language, memory, perception, attention, and control are cognitive capacities of interest, and moreover that these capacities deserve a separate place in our ontology. Thus, cognitive ontology concerns the question of what are the cognitive capacities that should be in our taxonomy of the mind. This is not merely a philosophical question, but a fundamental question for cognitive (neuro)science, because the field *presupposes* a particular cognitive ontology as a starting point for empirical research.

In the cognitive neuroscience literature, the term “ontology” gained momentum after a paper by Price & Friston in 2005, because they believed that traditional cognitive ontologies did not do justice to neuroscientific findings: “We will suggest that the advent of functional neuroimaging speaks to a neurobiologically informed ontology that is constrained and “boot-strapped” by our increasing knowledge of functional anatomy” (Price & Friston, 2005, p. 263). Now 20 years later, we can clearly see that we did not reach this ambitious goal. There is still no consensus on what is the right taxonomy for understanding cognition, nor for the domains of language and memory—reflected by the EWCN symposium and this featured article—nor for the field of cognitive neuroscience as a whole.

### 5.2 Neuroscience and cognitive ontology revision

Why is such consensus still lacking? There are at least four reasons why it turns out to be more difficult than expected to revise our cognitive ontology (for more comprehensive considerations, we refer the reader to the literature cited in this section), two of which will be discussed in this Section 5.2. First, we have the problem of “many-to-many mappings” in cognitive neuroscience (Francken & Slors, 2014; Janssen, 2019; Khalidi, 2017; Klein, 2017; McCaffrey & Machery, 2016; Sullivan, 2017). In the past decades, we have discovered that most of our cognitive capacities, such as attention or memory, do not map to the brain in a straightforward way. On the one hand, many brain areas perform many different cognitive functions, interact with other brain areas, and their activity/involvement is often context- or task-dependent. On the other hand, performance of a cognitive function often involves neural activation in different brain areas or even across the entire brain. The combination of these two characterizations results in a many-to-many mapping between cognitive capacities and the brain. As a consequence, it turns out to be difficult to characterize what regions of the brain actually do and consequently, to use neuroscientific knowledge to inform the cognitive ontology project.

A second reason why we still don’t know what is the right ontology for cognition results from the fact that there is no consensus in the scientific community about the *extent* to which our cognitive ontology should be revised as a result of neuroscientific insights. Anderson (2015) distinguishes three positions: conservatives, moderates and radicals. In brief, conservatives expect that limited revision of the cognitive ontology will suffice for a cleaner (one-to-one) mapping. As an example, Anderson discusses Price and Friston (2005) who propose to consider neurobiological evidence during analysis and decomposition of a cognitive process into a set of fundamental operations. The moderate position also expects that ultimately one-to-one mappings will be possible. Yet in contrast to conservatives, they believe that brain data is crucial to determine whether our current cognitive categories should be reconsidered. For instance, neuroscience might show that two putatively separate capacities should be unified (lumping) or even that a cognitive capacity should be eliminated from the cognitive ontology (see e.g., Lenartowicz et al., 2010). Finally, Anderson describes the radicals, who believe we should expect very few one-to-one mappings between brain and cognition. Moreover, they contend that we need to construct an ontology of very different kinds of categories than we currently have, and that are in some way “brain-derived”. Anderson discusses Poldrack et al. (2009) as an example, who propose six non-standard psychological dimensions in order to accurately predict brain activation patterns.

**Figure 6.**
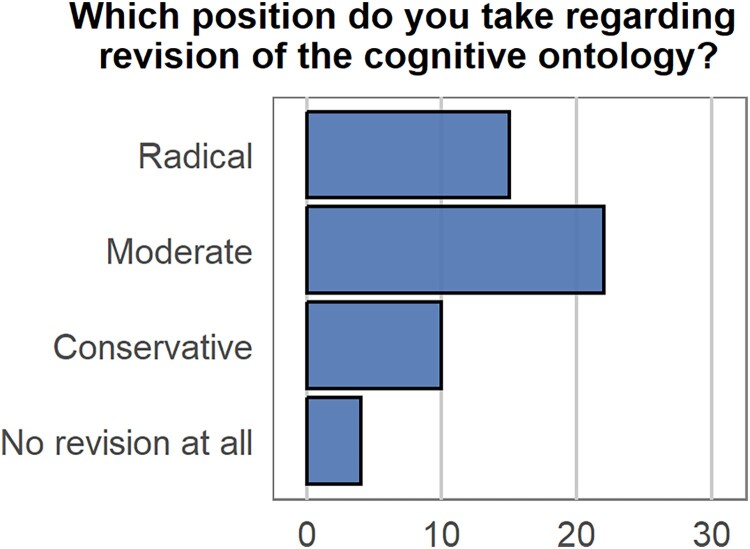
Poll response counts for each option for the prompt: “Which position do you take regarding the revision of the cognitive ontology?”. [To view this figure in colour, please see the online version of this journal.]

During the symposium at EWCN we surveyed the audience about their position regarding revision of the cognitive ontology (Figure 6). The distributed nature of the responses strengthens our argument that there is currently no consensus about the extent to which our cognitive ontology should be revised and in particular, about the proper role of neuroscientific evidence in the revision process.

Interestingly, all three positions—and the large majority of our EWCN audience—see a positive role for neuroscientific evidence in revision of the cognitive ontology, and as Anderson notes, urge us to reject the autonomy of psychology from neuroscience. This has been a debated issue since the 1980s, peaking in the early days of fMRI research (Coltheart, 2002; Fodor, 1974; see for a more recent, influential paper, Krakauer et al., 2017). It is worth mentioning one specific occasion of this debate, since it took place in Bressanone at EWCN in 2005. Here, Max Coltheart presented a paper titled “What has functional neuroimaging told us about the mind (so far)?” (Coltheart, 2006) in which he questioned the evidential status of neuroimaging data within cognitive psychology compared to behavioural measures.

Therefore, we do think that it is necessary to explore whether a fundamental assumption of all three revisionist positions—that it is possible to use neuroscientific data as an objective arbiter for cognitive similarity—is justified. Otherwise, the neuroscientific results as discussed in Sections 2–4 might be rendered irrelevant for specifying the relationship between the domains of language and memory.

### 5.3 Three interrelated problems for cognitive ontology revision

**Figure 7.**
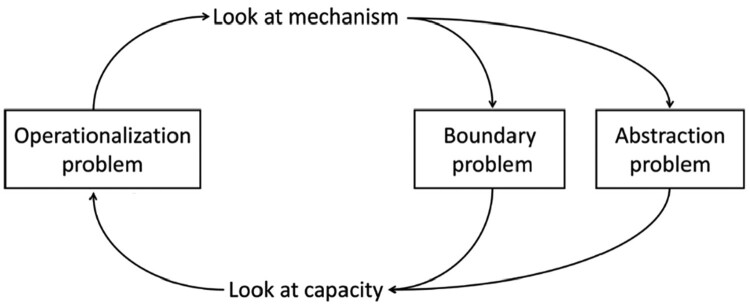
An overview of three interrelated problems for cognitive ontology revision. Figure reprinted from Francken et al. (2022b, Figure 1) with permission, https://creativecommons.org/licenses/by/4.0/. No changes were made.

The revisionist positions described by Anderson (2015) all seem to suppose that we can use neuroscientific data to inform us about underlying neural mechanisms for cognitive ontology revision. In a recent paper, Francken et al. (2022b) argue that this simple bottom-up revision approach is idealized. They demonstrate that the project of cognitive ontology revision faces three interrelated problems (Figure 7).

At the left side of Figure 7 we see the Operationalization problem (see also Feest, 2005, 2020; Sullivan, 2010, 2016): how to justify that a behavioural task measures the cognitive capacity it aims to measure? The Operationalization problem arises because we cannot observe the cognitive capacity independently of our choice of task. Now we could assert that the variety of tasks that we use to manipulate and measure e.g., episodic memory all measure the same thing: episodic memory. But whether in fact they do so is an empirical question. In psychology, this issue has traditionally been addressed by assessing a test’s construct validity (Cronbach & Meehl, 1955). In cognitive neuroscience, we might have a different strategy at our disposal: determine whether or not these different tasks elicit the same neural mechanism. That brings us to the right side of Figure 7. To determine that two tasks (e.g., free recall and cued recall) measure the same cognitive capacity (e.g., episodic memory), we want to use neural mechanisms as objective arbiters of cognitive similarity. When two tasks elicit the same neural mechanism, we conclude that they measure the same cognitive capacity. However, as Francken, Slors and Craver note, the mechanistic structure of the world is not simply perceived as such. Hence, two further problems arise here, that ultimately cycle back on themselves.

First, we encounter the “Abstraction problem” (Figure 7; see also Craver, 2009; Levy & Bechtel, 2013). To identify neural mechanisms as such, we need to abstract away from the complex network of causal connections in the brain. However, at one degree of abstraction two neural mechanisms might be not of the same kind, while when abstracted further, both fall under the same kind of mechanism. For example, as discussed in Section 4, spatial overlap based on metabolic demands derived from fMRI might lead to the conclusion of a shared mechanism for language and memory. However, if these metabolic demands underlyingly have two different electrophysiological responses, one would arrive at the conclusion that the mechanisms are not shared. Thus, the Abstraction Problem occurs because there is no uniquely correct degree of abstraction for describing any given system. Looking more or less abstractly at the mechanisms, we might lump and split one and the same mechanism differently. Second, neural mechanisms do not come neatly packaged for us—we need to distinguish constituent parts of mechanisms from their background conditions. For example, pre-stimulus decreases in power in the alpha range can be considered part of the neural mechanism of word retrieval (see Section 4), but also as enabling conditions that are not part of this mechanism (e.g., Griffiths et al., 2019). So again, one can be led to lump or split neural mechanisms differently depending on where we draw the boundaries—which entanglements one decides to include in the mechanism. This is what Francken, Slors and Craver call the Boundary Problem (Figure 7; see also Craver, 2007, 2009; Craver et al., 2021).

Importantly, these two latter problems can be resolved only by going back to our prior understanding of what the relevant cognitive capacities are and of how they are elicited in behavioural tasks. In other words, Francken et al. (2022b) show that we arrive back at the Operationalization problem: what counts as a neural mechanism depends on how we have specified the cognitive capacity to be explained to begin with. From this discussion it becomes clear that we should not expect a simple bottom-up, brain-driven reform of our cognitive ontology.

**Figure 8.**
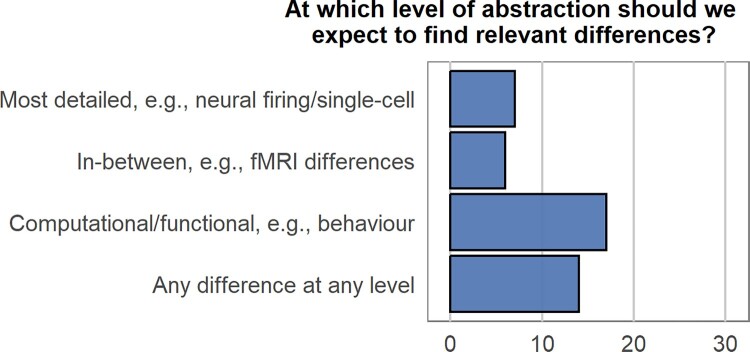
Poll response counts for each option for the prompt: “At which level of abstraction should we expect to find relevant differences that allow us to infer the relationship between the domains of language and memory?”. [To view this figure in colour, please see the online version of this journal.]

To show that the conceptual point of Francken et al. (2022b) is actually relevant for empirical research and the interpretation of neuroscientific data—in particular to determine to what extent language and memory are related domains—we surveyed the audience about the Abstraction problem: At which level of abstraction should we expect to find relevant differences that allow us to infer the relationship between the domains of language and memory? (Figure 8). Interestingly, again there was a clear lack of consensus among respondents. In conclusion, before we can start using empirical data to inform an answer to this question, conceptual discussion in the community seems to be required about the *type of evidence* that would be called for.

Apart from this (arguably limited) substantiation of one of the problems described by Francken et al. (2022b) by surveying the neuroscience audience at EWCN, it would be interesting to address in a more rigorous way—and preferably in dialogue with neuroscientists—whether the three problems as well as the model of iterative progress they propose resonates with the relevant scientists. Questions for future research could include: Does their model provide an accurate reflection of research practices in cognitive neuroscience? How are the three problems currently dealt with? Is the model of iterative progress testable, and should it be tested?

### 5.4 Is it reasonable to expect finding the right cognitive ontology?

So far, we have discussed three arguments for why we currently do not know what is the right cognitive ontology: the many-to-many mapping between cognitive capacities and the brain; the lack of consensus about the extent to which our cognitive ontology should be revised; and the fact that cognitive ontology revision is not a simple bottom-up process. Here, we would like to briefly highlight a possible fourth reason: searching for the *right* cognitive ontology is motivated by the wrong question.

Scholars from different academic disciplines have argued that our cognitive ontology is not as objective, scientific, universal, theory-neutral and ahistorical as we tend to think (see e.g., Danziger, 1997; McGeer, 2021; Uttal, 2003). Three examples may illustrate this apparently bold claim. First, we investigate e.g., “motivation” because this happens to be a relevant category in our Western culture and society rather than a universal one (Danziger, 1997). Second, it has been shown that many of our current cognitive ontology categories (e.g., learning, intelligence, personality) were “invented” or given radically different meanings in the twentieth century (Danziger, 1997). Thirdly, the introduction of new ontological categories and their subdivision can be shown to be highly influenced by technological development, such as statistical factor analysis or the introduction of neuroimaging tools (Curry, forthcoming; Uttal, 2003).

Although a full discussion of the literature is beyond the scope of this paper, we would tentatively suggest that assuming the existence of a single, true, cognitive ontology (“taxonomic monism”; McCaffrey & Wright, 2022) might set the bar too high and might ultimately even hinder progress. Alternatively, we may relax this assumption and embrace a more context- or goal-dependent, pluralist notion of cognitive ontology (Danks, 2015; Hochstein, 2016; McCaffrey & Wright, 2022; Sullivan, 2017). This latter approach would allow for a more nuanced answer to the question of how the domains of language and memory are related.

## 6. How to make progress

Although many in the field agree that understanding how language and memory are related is fruitful (Duff et al., 2020; Madan, 2021, 2024; Piai et al., 2016; Piai & Zheng, 2019; Roger et al., 2022), Section 5 illustrates this is easier said than done. From the empirical evidence, both in the literature and that briefly reviewed in Sections 2–4, we can infer that there is some kind of relation between episodic memory retrieval and word retrieval, and by extension, the domains of language and memory.

In this final section, we will integrate the empirical and philosophical perspectives and critically analyse what would be needed for the field to make claims about the relationship between the domains of language and memory. It will be clear from our argument that empirical research, combined with parallel conceptual development, will allow us to move forward.

### 6.1 Specification of “language” and “memory”

In this paper, we have attempted to keep the discussion focused on episodic memory retrieval and word retrieval to provide a more in-depth analysis of the issues that arise when assessing the relationship between language and memory. In general, an obvious, and relatively easy, first step to make progress is to clearly specify and define the cognitive capacities under study. Often, definitions of “language” and “memory” vary to some extent between studies and research groups. As a consequence, by starting off with different conceptualizations, researchers run the risk of investigating slightly different phenomena even though the same overarching label is used (e.g., “language”) (Figdor, 2013; Francken & Slors, 2014). To avoid this, we suggest not just defining cognitive capacities by stating scientific sub-concepts of language and memory, for example, episodic memory, linguistic memory, etc., but also by making explicit the operational definition of these terms in the specific experimental context (see also Section 5.3 on the Operationalization problem). A shared vocabulary at both conceptual and operational definition levels will benefit integration of empirical findings and avoid miscommunication.

### 6.2 Specification of the relationship between language and memory

During the discussion at the EWCN Symposium, different kinds of relationships between language and memory were mentioned, and interestingly, people were not always aware of the fact that they were referring to different kinds of relationships. So, if we ask the question how to slice the domain-cake, what is the actual relationship that we should establish to be able to answer it? Below we list some possibilities:

− Is language *the same as* memory?
− Is language *intertwined with* memory?
− Is language *a kind of* memory?
− Is language *part of* memory (or vice versa)?
− Is language *dependent on* memory (or vice versa)?
− Does language *(causally) influence* memory (or vice versa)?

This (incomplete) list exemplifies that, in our view, there are at least two issues that need to be addressed. First, there may exist *different kinds of relationships* between cognitive domains, and moreover we are not always aware of the fact that these are different. Second, people might *interpret one specific kind of relationship in different ways*, for instance, “language is part of memory” could mean that language is a specific kind of memory, but alternatively, it could also mean that the neural correlates of a specific language process and a memory process overlap.

In the literature, both of these ambiguities are present. For instance, above, in Section 4, we claimed that “the neuronal infrastructure people utilize for retrieving linguistic and episodic information is the same”. As another example, Renoult et al. (2019) write that “episodic and semantic memory are inextricably intertwined, yet retain a measure of distinctiveness, despite the fact that their neural correlates demonstrate considerable overlap”.^5^ Thus, we are not only confusing different kinds of relationships at the same level (e.g., cognitive level), but also mixing up inter-level relationships (e.g., cognitive and neural level). The latter is particularly problematic in light of the three interrelated problems described in Section 5.3. To answer the question of how language and memory are related in a constructive way, we first need to specify the relationship that we presuppose between the domains.

### 6.3 How to weigh different types of evidence?

The quotes above highlight a further important question: how to weigh different types of evidence, i.e., from neuropsychology vs. cognitive neuroscience, or from fMRI vs. neurophysiological studies, etc.?

**Figure 9.**
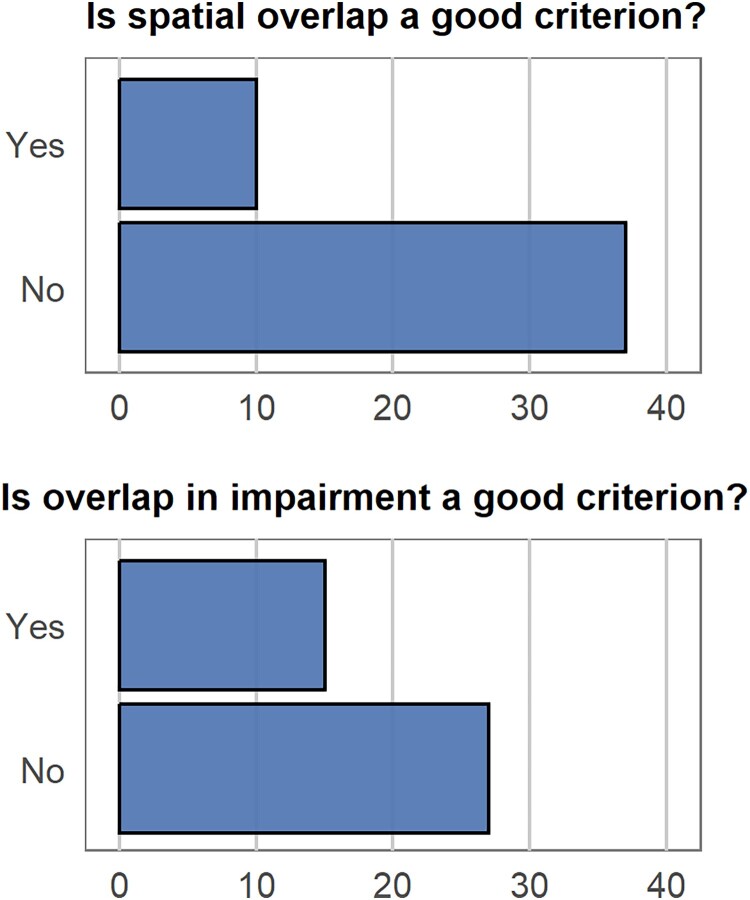
Poll response counts for each option for the prompt “Is spatial overlap a good criterion for claiming that episodic memory and lexical memory are the same domain?” (upper) and “Is overlap in impairment in episodic memory and lexical memory a good criterion for claiming that episodic memory and lexical memory are the same domain?” (lower). [To view this figure in colour, please see the online version of this journal.]

Since we were interested in knowing how researchers think about this issue, we surveyed the symposium audience on two specific types of evidence: overlap in the spatial dimension (e.g., using fMRI, there is spatial overlap between word retrieval and episodic memory retrieval effects, Figure 9, upper), and overlap in functional impairment (i.e., an association, e.g., both episodic memory and word retrieval are impaired after brain damage, Figure 9, lower). There was no unanimous opinion for either type of evidence and, in fact, the majority of respondents thought that in both cases overlap/association was not a good criterion.^6^ This is surprising, given the reliance of the field on these types of evidence. Moreover, if researchers use different criteria when integrating evidence, they will likely end up with different answers to the domain-cake question while basing their inference on the exactly same empirical data.

To increase opportunities for consensus, it may be informative to disentangle what one could refer to when using the phrase “types of evidence” in this context:

− Levels: brain or behavior or cognition;
− Measurement techniques: for example, fMRI, electrophysiology, lesion-symptom mapping, etc.
− Similarity or difference: association vs. dissociation (lesion-symptom studies) or overlap vs. non-overlap (brain areas, neurophysiological responses)
− Convergence: is one type of evidence sufficient or is convergence across methods necessary/superior (e.g., by triangulation, Lawlor et al., 2017; Vaidya et al., 2019)

We believe a discussion in the scientific community about how to weigh different types of evidence would benefit from disentangling these (partly related) aspects.

Second, we need to ask this question in light of the specific kind of relationship that we are aiming to establish (see Section 6.2), i.e., what type of evidence is needed for supporting a particular kind of relationship? For example, if one wants to speak to the causal relationship, one may need a different type of evidence than if one wants to claim a “part-relationship”. Moreover, certain types of evidence, and of “similarity” in particular, may demand a higher level of evidence than that of “difference”.^7^

Furthermore, the philosophical discussion above (Section 5) shows that there are at least two important background assumptions that need to be made explicit when weighing different types of evidence. First, we should give a justification for the relevance of the degree of abstraction of the evidence, and second, we should make explicit our revisionist position: In case we would acquire the specific evidence, would we be willing to use it to revise our current cognitive ontology, i.e., the cognitive categories themselves and the boundaries we draw between (sub)categories of language and memory?

### 6.4 Critical re-analysis and evaluation of empirical claims

Here, we will attempt to evaluate one of our own claims following the critical discussion presented so far in Section 6. In Section 4 above, we presented the finding that *the neurophysiological effects (a proxy for the neuronal operations) for word retrieval and episodic-memory retrieval were similar*. Would this piece of evidence justify a claim about one of the possible relationships between the domains (see Section 6.2), for example, that language and memory are the same domain?

Following the characterization of the type of evidence discussed in Section 6.3, the “measurement technique” in this case is fixed by the type of neurophysiological responses on which the empirical claim is based, with the assumption that this measurement technique measures at a relevant degree of abstraction. Second, regarding “similarity or difference”, the evidence is one of similarity/association/overlap, with the assumption that the operationalizations being used are sufficiently similar (and not confounded). Next, regarding “convergence”, we are using one specific piece of evidence for this claim, assuming that this is sufficient to justify our inference. Finally, regarding the “levels” at which the evidence is located, we use brain data for a claim about cognition/cognitive processing, thus assuming that neural evidence is relevant for claims about the relatedness of cognitive functions. This assumption could be interpreted as displaying a moderate revisionist position (see Section 5.2). Note that none of these assumptions were made explicit originally (Piai & Zheng, 2019; Section 4), nor are they supported by arguments.

Given that we agree that neural evidence is relevant for claims about cognition, what kind of relationship between language and memory can we infer on the basis of this piece of evidence? Readers will probably agree that the inference that “language and memory are the same domain” is too strong. Yet noticeably, we come to the realization that determining a more specific kind of relationship between the two domains is not possible on the basis of the particular evidence discussed here. In fact, multiple if not all kinds of relationships that we listed above are compatible with this piece of evidence. Likewise, the relationship of intertwinement, as per “language is intertwined with memory” is (almost) always true, and as such, perhaps less useful as a description of the relationship between the two domains. Similarly, the relationship we claimed above in Section 2 “language is based in memory” can also be specified further.

In conclusion, finding similar neurophysiological effects for word retrieval and episodic-memory retrieval does not warrant the claim that language and memory are the same domain, nor does it warrant general claims about any of the other kinds of relationships. The re-analysis and evaluation presented here shows us our implicit assumptions and the extent to which our claims are justified by the evidence, and points to the need to define explicitly and a-priori the target kind of relationship one wants to understand. That is, the kind of relationship one wants to make claims about will determine the type of evidence needed, and consequently, the type of experimental design and approach.

### 6.5 Concluding remarks

The discussion presented here shows that it is unsurprising that we currently do not know how to slice the domain cake—or what the nature of the relationship between language and memory is. We have discussed several reasons for why answering these types of questions is more complicated than one might suppose. Some of these reasons are a consequence of the complexity of our objects of study—cognitive capacities such as language and memory, and neural mechanisms—but other reasons are conceptual in nature. That means that progress can be expected not just by collecting more empirical data, but requires parallel conceptual development.

Here we have analysed several conceptual issues that might contribute to such progress. Note that similar issues are being discussed in other fields of cognitive (neuro)science. For instance, in the neuroscience of consciousness, a recent survey showed that researchers have different objectives and pursue them by using a variety of methods (Francken et al., 2022a). In the field of memory, it has been recognized that it is critical to align the interdisciplinary understanding of concepts such as learning or retrieval (Roediger et al., 2007). Importantly, we do not recommend that the field must reach a consensus on, for example, the definition of language, the “right” degree of abstraction, or the “best” measurement technique. Rather, we propose that formulating the precise aim of the experiment, in a particular context, already is likely to lead towards more specific answers to these kinds of questions. Making explicit assumptions that accompany these decisions will probably show that there are multiple different, valid ways to study language, memory, and their relationship.

Here we have proposed to reconsider the question of how to see the relationship between the domains of language and memory to generate more productive answers. With this interdisciplinary perspective—from cognitive psychology, cognitive neuropsychology, cognitive neuroscience, and philosophy of neuroscience—we hope to contribute to scientific progress in the field by suggesting where and how to look for answers, but also by critically analysing the questions themselves.

## Notes

1 Interestingly though, it has been shown that current distinctions between cognitive domains such as language and memory have not always been drawn in the same way in the past, see e.g., Uttal (2003) tracing back our so-called “cognitive ontologies” and Danziger (1997) for demonstrating the historicity of our cognitive concepts. We discuss this issue in more detail in Section 5.

2 The survey was conducted using Wooclap. Survey participants consented to their data being used for the poll answers we present here. Participants could walk in and out of the 3-hours symposium at any time and could choose when to engage in the poll. So 89 is the maximum number of symposium attendees in total who registered to participate at any point in time.

3 There are of course limitations to picture naming as a task for this purpose—as there are, in fact, to any one particular task used in isolation.

4 See Hilverman and Duff (2021) for discussion of these findings in terms of impaired “maintenance of lexical-conceptual-object bindings” vs impaired retrieval. Our loose definition of word retrieval above is compatible with both of these interpretations.

5 We do not intend to be overly critical of these authors. We too have previously made comparable claims in other contexts (e.g., Madan, 2020; Piai & Zheng, 2019).

6 Note that we did not ask about non-overlap, so we cannot extend these poll results to have implications about non-overlap, but see the Abstraction problem question in Section 5.3.

7 We thank Brad Mahon for raising this important point.
